# Sustained inactivation of the Polycomb PRC1 complex induces DNA repair defects and genomic instability in epigenetic tumors

**DOI:** 10.21203/rs.3.rs-4289524/v1

**Published:** 2024-04-24

**Authors:** Chetan C. Rawal, Vincent Loubiere, Nadejda L. Butova, Juliette Garcia, Victoria Parreno, Anne-Marie Martinez, Giacomo Cavalli, Irene Chiolo

**Affiliations:** University of Southern California; CNRS, University of Montpellier; University of Southern California; CNRS, University of Montpellier; CNRS, University of Montpellier; CNRS, University of Montpellier; CNRS, University of Montpellier; University of Southern California

**Keywords:** Epigenetically initiated cancers, Polycomb complex, Double-strand break repair, Genomic instability

## Abstract

Cancer initiation and progression are typically associated with the accumulation of driver mutations and genomic instability. However, recent studies demonstrated that cancers can also be purely initiated by epigenetic alterations, without driver mutations. Specifically, a 24-hours transient down-regulation of polyhomeotic (ph-KD), a core component of the Polycomb complex PRC1, is sufficient to drive epigenetically initiated cancers (EICs) in Drosophila, which are proficient in DNA repair and are characterized by a stable genome. Whether genomic instability eventually occurs when PRC1 down-regulation is performed for extended periods of time remains unclear. Here we show that prolonged depletion of a PRC1 component, which mimics cancer initiating events, results in broad dysregulation of DNA replication and repair genes, along with the accumulation of DNA breaks, defective repair, and widespread genomic instability in the cancer tissue. A broad mis-regulation of H2AK118 ubiquitylation and to a lesser extent of H3K27 trimethylation also occurs, and might contribute to these phenotypes. Together, this study supports a model where DNA repair and replication defects amplify the tumorigenic transformation epigenetically induced by PRC1 loss, resulting in genomic instability and cancer progression.

## Introduction

The tumorigenic process is typically associated with DNA damage defects and genomic instability (Hopkins, Lan and Zou 2022). However, recent studies established that cancer can also be driven purely by epigenetic changes initiated by the transient loss of the transcriptionally repressive Polycomb PRC1 complex (Parreno 2024). Polycomb Group (PcG) proteins are grouped in two main classes of complexes called Polycomb Repressive Complex 1 and 2 (PRC1 and PRC2) (Levine, Weiss et al. 2002, Kassis, Kennison and Tamkun 2017, Schuettengruber, Bourbon et al. 2017). *Drosophila* PRC1, which is composed of PH, PC, PSC and SCE subunits, is primarily responsible for H2AK118 ubiquitylation (H2AK118ub, or H2AK119ub in mammals) (Barbour, Daou et al. 2020, Parreno, Martinez and Cavalli 2022), whereas PRC2 mediates H3K27 trimethylation (H3K27me3) (Holoch and Margueron 2017). PRC1 and PRC2 are also highly interdependent, given that PRC1 binds to H3K27me3, and PRC2 associates with H2AK118ub (Blackledge, Farcas et al. 2014, Cooper, Grijzenhout et al. 2016, Kasinath, Beck et al. 2021). This enables cooperative binding of the two complexes to the same sites and codependency in the establishment of the respective marks on chromatin (Cooper, Grijzenhout et al. 2016, Barbour, Daou et al. 2020, Tamburri, Lavarone et al. 2020). While PRC1 and 2 form several redundant sub-complexes in mammalian cells (Parreno, Martinez and Cavalli 2022, de Potter, Raas et al. 2023), *Drosophila* Polycomb complexes comprise a reduced number of paralogous and accessory subunits, facilitating the study of these components in flies.

PRC1 and PRC2 co-regulate a variety of cellular processes including embryonic development, differentiation, and cell proliferation (Chan and Morey 2019, Loubiere, Martinez and Cavalli 2019, Loubiere, Papadopoulos et al. 2020). Consistent with a role for PcG proteins in cell identity, dysregulation of these components has been associated with multiple types of cancer (Piunti and Shilatifard 2021), including breast and prostate cancers, as well as hematologic malignancies (Varambally, Dhanasekaran et al. 2002, Guo, Zeng et al. 2007, Li, Li et al. 2010, Zhang, Sheng et al. 2010, Ntziachristos, Tsirigos et al. 2012, Herviou, Cavalli et al. 2016, Kim and Roberts 2016, Althobiti, Muftah et al. 2020). In agreement, PRC1 loss-of-function mutations in *Drosophila* result in up-regulation of major oncogenes including JAK/STAT, NOTCH and JNK signaling pathways, which are important drivers of the tumorigenic process (Classen, Bunker et al. 2009, Martinez, Schuettengruber et al. 2009, Loubiere, Delest et al. 2016, Torres, Monti et al. 2018).

Recent studies also suggest a role for PRC1 in DNA double-strand break (DSB) repair by homologous recombination (HR) and, to a lesser extent, non-homologous end joining (NHEJ) (Vissers, van Lohuizen and Citterio 2012). Upon exposure to ionizing radiation (IR) or FokI-induced DSBs, PRC1 core subunits are quickly and transiently recruited to the damage site in an ATM-dependent manner, where they induce H2A/H2AX K119ub and transcriptional silencing (Kakarougkas, Ismail et al. 2014, Kakarougkas, Downs and Jeggo 2015, Ui, Nagaura and Yasui 2015). This histone modification also promotes the recruitment of DSB repair components including 53BP1, BRCA1, RAP80, and the resection protein CtIP (Ismail, Andrin et al. 2010, Pan, Peng et al. 2011, Ismail, McDonald et al. 2013, Kakarougkas, Ismail et al. 2014, Fitieh, Locke et al. 2021, Fitieh, Locke et al. 2022). However, the extent to which DSBs strictly relies on PRC1 for repair remains unclear, as loss of this component only partially affects repair kinetics and the resulting sensitivity to IR exposure is modest (Ismail, Andrin et al. 2010, Fitieh, Locke et al. 2021).

Importantly, even a transient depletion of PRC1 core complex subunits leads to cancer formation in *Drosophila* (Parreno 2024). Specifically, a 24-hr depletion of the PRC1 subunit PH results in irreversible activation of key members of the JAK-STAT pathway, which in turn triggers a switch to a self-sustaining cancer cell fate, even upon restoration of normal PRC1 activity (Parreno 2024). These EICs are proficient in DSB repair and do not show chromosome rearrangements or major increase in the mutational load (Parreno 2024).

Here we investigate whether a sustained inactivation of PRC1, which mimics a cancer-inducing context, eventually results in DNA damage repair defects and genomic instability. We show that inactivation of PH over 5 days is sufficient to induce massive over-replication, the mis-regulation of several repair genes, and a broad reduction in H2AK118ub and H3K27me3. Consistently, these tumors have elevated levels of endogenous DNA damage, DSB repair defects, and massive genomic instability. Together, these results are consistent with a model where EICs derived from transient PcG inactivation can rapidly transition to a state characterized by a highly genetically unstable genome. This instability might further contribute to tumor development when Polycomb depletion is maintained.

## Materials And Methods

### Drosophila strains, genetics, and growth conditions

**Figure 1:**
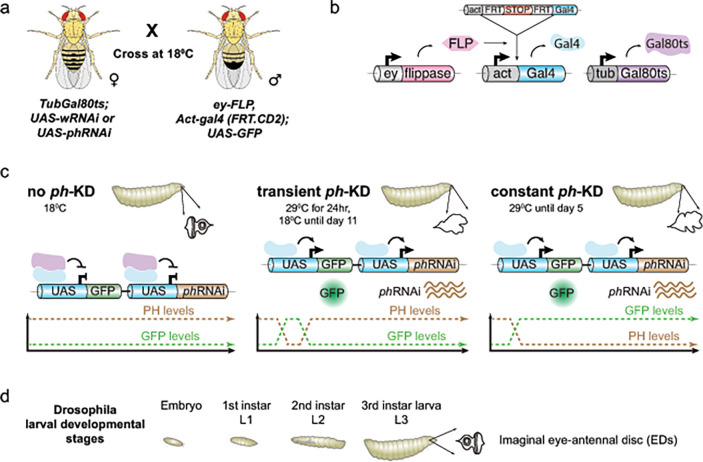
Schematic representation of the experimental setup used to induce PRC1-dependent Cancers. **a)** Scheme of the experimental cross used to generate progeny with thermosensitive conditional knockdown using *phRNAi* or *whiteRNAi*. Female virgins from the fly line with *ey-FLP, Act-gal4, UAS-GFP* were crossed with males from the fly line with tub-Gal80ts and *UAS-phRNAi* or *UAS-wRNAi* at 18°C. **b)** Expression of the flippase *ey*-FLP (pink) in imaginal eye-antennal disc cells catalyzes the FLP-out of a transcriptional stop (red) in the developing discs, allowing the expression of *act*-Gal4 (light blue). Constitutively expressed *tub*-Gal80ts (purple) encodes a temperature-sensitive Gal4 repressor. **c** At 18°C, TubGal80ts inhibits Gal4-mediated *phRNAi* as well as *GFP* expression (used as internal control), thereby maintaining high levels of PH, leading to normal ED development **(d)**. Shift of the developing embryo/larvae to the restrictive temperature of 29°C for 24 h or 5 d leads to transient or constant *ph*-KD, respectively, thereby inducing tumors, which can be dissected at the 3rd instar stage of larval development.

*Drosophila* flies were maintained on a standard corn-meal yeast extract medium at 25°C, unless otherwise indicated. Crosses were performed as described in (Parreno 2024) (See also Fig. 1). Briefly, Gal80ts was used to achieve complete depletion of PHor the control *white* gene by switching the temperature from 18°C to 29°C. The *ey*-FLP system was used to generate complete knockdowns in the larval eye-antennal imaginal discs (EDs). Flies were reared and crossed at 18°C to inhibit Gal4 activity. 6 independent crosses were set up using 80 virgin females with 20 males for each genotype and egg laying was carried out for 4 hours at 18°C to synchronize the embryonic and larval development. For achieving constant *ph*-and *white*-KD, tubes containing eggs were immediately shifted to 29°C throughout the development, and third instar larvae were dissected 5 days after egg laying (AEL). Embryos maintained at 18°C throughout and dissected 11 days AEL were also used as control (no *ph*-KD). The genotypes of the flies which were used to Knock-Down (KD) *ph* or *white* are as follows: For control *white*-KD: *ey-FLP, Act-gal4 (FRT.CD2 STOP)* (BL#64095); *TubGal80ts* (BL#7019); *UAS-wRNAi* (BL#33623)/*UAS-GFP* (BL#64095). For *ph*-KD: *ey*-*FLP, Act-gal4 (FRT.CD2 STOP)* (BL#64095); *TubGal80ts* (BL#7019); *UAS-phRNAi* (VDRC#50028)/*UAS-GFP* (BL#64095).

### Immunostaining and fluorescence microscopy

Third instar female larval heads were dissected to isolate eye-antennal imaginal discs (EDs) at RT in 1x PBS. Tissues were fixed in 4% formaldehyde for 30 minutes on a rotating wheel. Permeabilization was carried out for 1 hour in 1x PBS containing 0.5% Triton X-100 on a rotating wheel. Blocking was performed for 1 hour using 3% BSA PBTr (1x PBS + 0.1% Triton X-100), and incubated with the primary antibody against phosphorylated Histone H2AvD (Rabbit anti-Histone H2AvD pS137, dilution 1:500 prepared in 1% BSA PBTr, Rockland, 600-401-914) for 2 hours at RT. Then, samples were washed in 1x PBTr for 15 minutes each for 3 times before adding a secondary antibody (Donkey anti-rabbit Alexa Fluor 488, dilution 1:1000 in 1% BSA PBTr, Invitrogen, A-21206) for 2 hours at RT, on a rotating wheel. Tissues were then washed in PBTr for 15 minutes each for 3 times prior to DAPI staining at a final concentration of 1 μg/mL for 15 minutes. The discs were briefly washed in PBTr and in 1xPBS for 5 minutes each. Discs were mounted in Vectashield medium (Eurobio scientific, catalog no. H-1000–10) or ProLong Gold antifade agent (Life Technologies, P36930). Images for quantification of DSB foci were taken with a DeltaVision deconvolution microscope using a 60x oil immersion objective (NA 1.42 oil) and a CoolSNAP HQ2 camera. Images were processed using deconvolution through SoftWoRx 6.0.

### EdU labeling to assess replication

Ethynyl-2′-deoxyuridine (EdU, thymidine analogue) labeling were performed using Click-iT Plus EdU Alexa fluor 555 Imaging kit (Invitrogen, #C10638) as per manufacturer’s instructions. The eye-antennal imaginal discs of female third instar larvae were dissected in Schneider medium and EdU was added at a final concentration of 25 μM on a rotating wheel at RT for 15 min. After washing with PBS, tissues were fixed in 4% formaldehyde 30 min and washed 3 times with PBS. The imaginal discs were permeabilized for 1h in 1xPBS + 0.5% Triton X-100 on a rotating wheel then blocked for 1h in 1xPBS + 0.1% Triton X-100 + 3% BSA. EdU detection was performed according to the manufacturer’s instructions for 30 min on a rotating wheel at RT away from light. 500 μl of Click-iT reaction solution was prepared per tube containing 10–12 eye-antennal imaginal discs. After 1xPBS + 0.1% Triton wash, DAPI staining was performed at a final concentration of 1 μg/ml during 15 min. Tissues were washes in 1xPBS + 0.1% Triton and discs were mounted in Vectashield medium. Image acquisition was performed using a Leica SP8-UV confocal microscope with a 10x objective (NA 0.4).

### Fluorescent In Situ Hybridization (FISH) to analyze karyotypes

Chromosome preparation and FISH was performed as previously described (Larracuente and Ferree 2015, Ryu, Spatola et al. 2015). Eye discs and tumors from L3 female larvae were dissected in 0.7% NaCl solution and were incubated in the Colchicine solution (3 ml of 0.7% NaCl + 100 μl of 10–3 M Colchicine) for 1 hour at RT away from light. Following Colchicine treatment, tissues were incubated in 0.5% Sodium Acetate for 7 minutes, and fixed using freshly prepared 2.5% PFA in 45% acetic acid for 4 minutes on a coverslip. Tissues were pressed onto poly-lysine coated slides using manual force and snap frozen in liquid nitrogen. The slides were washed in 100% ethanol for 5 minutes, air dried and stained with FISH probes for AACAC, AATAT and 359-bp repeats, as previously described (Larracuente and Ferree 2015). Probe sequences are: 5′–6-FAM-(AACAC)7, 5′-Cy3-TTTTCCAAATTTCGGTCATCAAATAATCAT, and 5′-Cy5-(AATAT)6. Microscopy acquisition was performed on a DeltaVision deconvolution microscope using a 60x oil immersion objective (NA 1.42 oil) and a CoolSNAP HQ2 camera. Images were processed for Deconvolution using SoftWoRx 6.0.

### Ionizing radiation exposure to induce DNA damage

L3 early-stage female larvae were transferred into a petri dish containing standard food medium and were irradiated with the dose of 5 Gy of X-rays using a Precision X-RAD iR160 irradiator. After irradiation, larvae were maintained in the petri dish at 29°C. Larval heads were dissected at indicated time points at RT in 1x PBS and fixed in 4% paraformaldehyde for 30 min, before immunostaining. Microscopy and image analysis were performed as described above. Due to accelerated pupation of L3 stage larvae at 29°C, DSB repair analysis was limited to 4 hours post-irradiation.

### Bioinformatic analyses

All in-house bioinformatic analyses were performed in R version 3.6.3 (URL: https://www.R-project.org/) and are publicly available at https://github.com/vloubiere/Rawal_et_al_HCB_2024.git. Computations on genomic coordinate files and downstream computations were conducted using the data.table R package (data.table: Extension of ‘data.frame’. https://r-datatable.com, https://Rdatatable.gitlab.io/data.table, https://github.com/Rdatatable/data.table, v1.14.2). In all relevant panels of Figures and Extended Data Figures, box plots depict the median (line), upper and lower quartiles (box) ±1.5x interquartile range (whiskers) and outliers are not shown. For each relevant panel, the statistical test that was used is specified in the caption, and the same abbreviations were used regardless of the test: n.s.= Not significant, *pval<5e-2, **pval<1e-2, ***pval<1e-3, ****pval<1e-5.

### ChIP-seq and CUT&RUN data analysis

ChIP-seq datasets and the processed data files were downloaded from GEO (GSE222193, (Parreno 2024)), and are listed Supplementary table 1. PH ChIP-seq and, H2AK118Ub and H3K27me3 CUT&RUN coverage was computed using 2.5kb bins covering all canonical chromosomes (X, 2L, 2R, 3L, 4), and were visualized using Hilbert curves (Anders 2009) and an iteration level of 10. To compute enrichment ratios around the TSS of PcG-bound genes (−25kb to +75Kb), H2AK118ub and H3K27me3 coverage was normalized to a set of activity-matched, unbound genes (n= 610 for each group).

### RNA-seq data analysis

RNA-seq datasets and the processed output files were obtained from GEO (GSE222193, (Parreno 2024)), and are listed Supplementary Table 1. Differential expression analysis output performed using the DESeq2 R package (Love, Huber and Anders 2014) (v1.26.0) were obtained from ((Parreno 2024), Extended Data Table 1).

### GO terms enrichment

Gene Ontology (GO) terms associated to genes that were up-regulated (padj<0.05 & log2FoldChange>1) or down-regulated (padj<0.05 & log2FoldChange>1) after constant or transient *ph*-KD were retrieved using the AnnnotationDbi R package (https://bioconductor.org/packages/AnnotationDbi.html, v1.48.0). For each GO term, over-representation was then assessed over a background set of genes – consisting of all the genes that passed DESeq2 initial filters – using a one-sided Fisher’s exact test (alternative = “greater”). Obtained p-values were corrected for multiple testing using False Discovery Rate (FDR).

Differentially expressed genes associated to “cellular response to DNA damage”, “DNA repair” and “DNA replication” GO terms are available in Supplementary Table 2, together with 6 other genes which were associated to the “cellular response to DNA damage stimulus”, which were nevertheless excluded from Fig. 3d due to the likelihood that their role in DNA damage response is indirect (Supplementary table 2).

### Human and Animal Rights

The study did not involve any human or animal subjects.

## Results

### Prolonged loss of the core PRC1 subunit PH (*ph*-KD) triggers genome instability

Recent studies showed that knocking down the PRC1 subunit PH for a short time (24 h, Fig. 1, transient *ph*-KD) during L1 larval stage is sufficient to induce EIC formation in third instar larvae (L3), and these EICs do not exhibit DNA repair defects or genomic instability (Parreno 2024). These studies used an efficient thermosensitive *ph*-RNAi fly system to acutely deplete PH with a 24 h incubation at 29°C, and normal levels were restored within 48 h after switching to 18°C(Parreno 2024). The same system was used to address the effect of prolonged PRC1 inactivation (constant *ph*-KD), thus enabling direct comparisons with transient *ph*-KD. Additionally, *white*-KD or larvae maintained at 18°C (no *ph*-KD) were used as controls (Fig. 1). Constant *ph*-KD was obtained by incubating the larvae at 29°C during the whole larval development for 5 days. Similar to transient *ph*-KD(Parreno 2024), constant *ph*-KD also results in tumor formation in 100% of eye-antennal imaginal discs (EDs) of L3 larvae (Parreno 2024).

### Prolonged *ph*-KD results in H2AK118ub and H3K27me3 loss at Polycomb target sites

**Figure 2:**
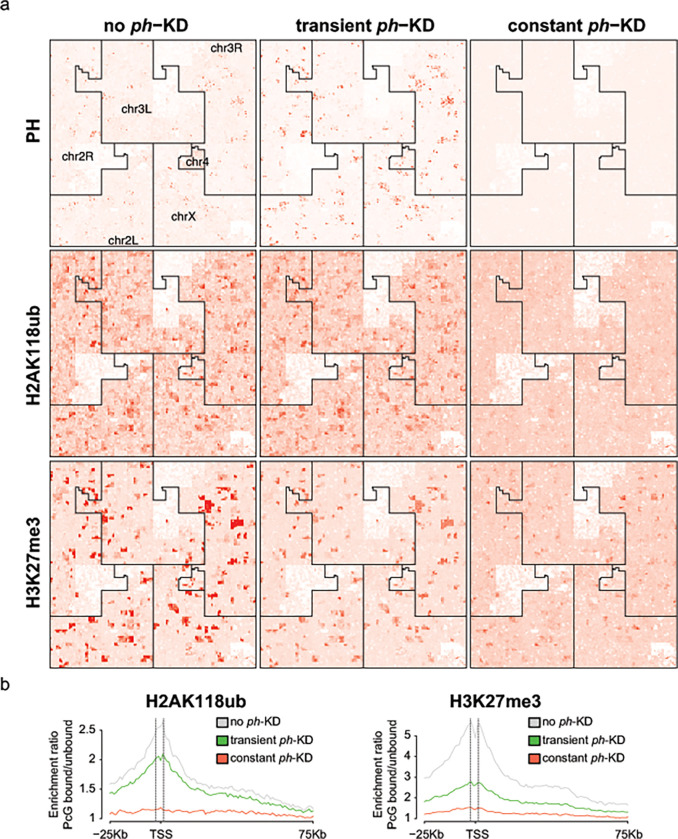
Tumors induced by constant *ph*-KD display loss of H2AK118ub and H3K27me3 at PcG target genes. **a)** Hilbert curve visualization of for PH ChIP-seq, H2AK118ub and H3K27me3 CUT&RUN in control (no *ph*-KD), transient *ph*-KD and constant *ph*-KD conditions. **b)** Enrichment ratios of H2AK118ub (left) and H3K27me3 (right) marks around the TSS (−25 to +75Kb) of PcG-bound genes compared to a control set of activity matched, PcG-unbound genes (n=610 genes for each group).

Given that both transient and constant *ph*-KD results in tumors characterized by loss of polarity and differentiation, we asked whether these tumors differ at the epigenetic level. We plotted the genome-wide enrichments of PH, H2AK118ub, and H3K27me3 from control eye-antennal discs (EDs, no *ph*-KD), EICs after transient (24 h) *ph*-KD and tumors obtained after constant (5 d) *ph*-KD, using published ChIP-seq and CUT&RUN data sets (GSE222193 from (Parreno 2024), Supplementary table 1). As shown in Fig. 2a, Hilbert curve plotting shows that PH expression and recruitment to chromatin are restored after transient *ph*-KD but not after constant *ph*-KD. Consistently, the analysis of H2AK118ub and H3K27me3 enrichments around PRC1 target genes (PRC1-bound) relative to PRC1 non-target genes (PRC1-unbound) shows that these modifications are largely restored after transient *ph*-KD, but not after constant *ph*-KD (Fig. 2b and Supplementary Fig. 1a). The most significant difference between EICs derived from transient *ph*-KD and constant *ph*-KD tumors is associated with H2AK118ub, consistent with this histone modification being the primary modification established by PRC1. We conclude that tumors resulting from prolonged *ph*-KD are characterized by extensive loss of H2AK118ub and H3K27me3 at PcG target genes, while this is not the case for EICs resulting from transient *ph*-KD.

### Prolonged *ph*-KD results in up regulation of DNA replication and DNA repair genes

**Figure 3:**
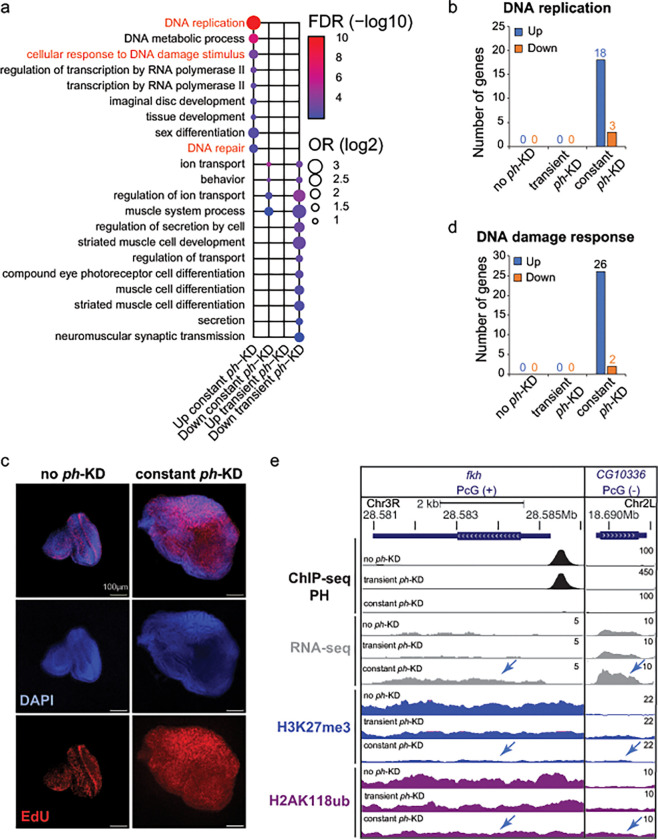
Contrary to EICs, tumors induced by constant *ph*-KD display dysregulation of DNA damage response and replication associated genes. **a)** Representative GO terms enriched in genes differentially expressed after constant or transient *ph*-KD (up or down regulation) (complete list available in Supplementary Fig. 1b) **b)** Number of genes specifically dysregulated after constant *ph*-KD representing GO term DNA replication. **c)** EdU and nuclear staining (DAPI) of ED imaged at day 11 for no *ph*-KD (control, left) and tumor at day 5 for constant *ph*-KD (right). **d)** Number of genes specifically dysregulated after constant *ph*-KD and associated to GO terms related to the DNA damage response. **e)** Genome browser snapshots for representative genes upregulated only in constant *ph*-KD conditions showing PH, H2AK118ub and H3K27me3 normalized tracks (ChIP-seq or CUT&RUN) and gene expression by RNA-seq, in control (no *ph*-KD), transient *ph*-KD and constant *ph*-KD conditions.

Given the major epigenetic differences between EICs generated by transient and constant *ph*-KD, we examined the differential gene expression between these tumors compared to control tissues (no *ph*-KD) and temperature-matched *white*-KD, using the published datasets derived from RNA-seq analyses (Parreno 2024). As shown in Fig. 3a and Supplementary Fig. 1b, we found significant differences in gene expression profiles between transient and constant *ph*-KD tumors.

Remarkably, gene clusters corresponding to Gene Ontology (GO) terms related to DNA replication, DNA damage and DNA repair were mostly up-regulated in constant *ph*-KD conditions relative to transient *ph*-KD tumors (Fig. 3a). Consistently, a fold-change analysis of all the genes classified as “DNA replication” (n=111) or “DNA damage response” (n=242) shows a significantly higher level of transcription for both categories in constant *ph*-KD tumors relative to control, and also compared to all genes (Supplementary Fig. 1c). This indicates that DNA replication and DNA damage response genes are overall more transcriptionally active in tumors derived from sustained *ph*-KD.

Within this general trend, 21 genes required for “DNA replication” and 28 genes required for the “DNA damage response” were the most affected, displaying at least a 2-fold change in expression specifically in constant *ph*-KD tumors relative to controls, most of which (18 and 26 genes, respectively) were up-regulated (Fig. 3b, d).

Most of the DNA replication and DNA damage response genes up-regulated in tumors derived from constant *ph*-KD are not associated with PRC1 enrichments in normal tissues (no *ph*-KD), suggesting that they are not direct targets of PH and their up regulation is an indirect effect of PRC1 loss (e.g., Fig. 3e, CG10336, or TIPIN in mammals). The most notable exception is the replication, repair, and transcription factor Fkh (FOXA2 and FOXA1 in mammals) (Knott, Peace et al. 2012, Li, Coic et al. 2012, Dummer, Su et al. 2016, Jin, Liang and Lou 2020, Hoggard, Hollatz et al. 2021), which is enriched for PRC1 in normal tissues, suggesting that PRC1 down-regulation directly affects the transcription of this gene (Fig. 3e).

The replication genes affected in constant *ph*-KD tumors include key replication components: the MCM complex, origin firing factors, and several DNA polymerases (Supplementary Table 2). This might result from an overall induction of replication in the tissue. Thus, we investigated the proliferation state of the cells by EdU incorporation and labeling in these tumors. As shown in Fig. 3c, control EDs show a few replicating cells, mostly localized at the morphogenetic furrow (Avellino, Peng and Lin 2023, Parreno 2024). Conversely, tumors derived from constant *ph*-KD are characterized by massive EdU incorporation, indicating a complete switch to an uncontrolled over-proliferating state (Fig. 3c). Of note, DNA replication-associated genes are found over-expressed also in transient *ph*-KD tumors (Supplementary Fig. 1c), albeit to a lesser extent compared to constant *ph*-KD tumors. Similarly, transient *ph*-KD tumors are also highly enriched for replicating cells (Parreno 2024).

Together, these results underscore that constant *ph*-KD leads to tumors characterized by the upregulation of several DNA replication genes, which is likely a consequence of cell hyper-proliferation. This up-regulation is more pronounced than that observed in EICs, and might reflect an even higher proliferation rate. Upregulation of components required for replication initiation and progression can also contribute to the acquisition of the hyper-proliferative state (Yu, Wang et al. 2020). In addition, we observed dysregulation of several DNA damage response genes upon constant depletion of PH, most of which are likely the indirect consequence of PH loss. These genes are mostly expressed at normal levels in transient *ph*-KD tumors, representing a major difference between the effects of short-term and long-term PH depletions.

### Prolonged *ph*-KD leads to defective DSB repair and increased genomic instability compared to EICs

DNA repair genes over-expressed in constant *ph*-KD tumors include several components previously linked to damage accumulation, cancer formation, and/or poor cancer prognosis (Table 1), like Mms4 (Dewalt, Kesler et al. 2014), RecQ4 (Maire, Yoshimoto et al. 2009, Su, Meador et al. 2010, Xu, Chang et al. 2021), PolH ( Tomicic, Aasland et al. 2014, Sonobe, Yang et al. 2024), Tipin/Timeless (Zhou, Zhang et al. 2020, Chen, Zhang et al. 2022), Claspin (Choi, Yang et al. 2014), MRNIP (Staples, Barone et al. 2016, Bennett, Wilkie et al. 2020, Wang, Zhao et al. 2022), FANCI (Smogorzewska, Matsuoka et al. 2007, Li, Yu et al. 2023), MMR proteins (Msh2, Mlh1, Msh6) (Shcherbakova and Kunkel 1999, Velasco, Albert et al. 2002, Li, Liu et al. 2008, Wagner, Webber et al. 2016, Wilczak, Rashed et al. 2017, Chakraborty, Dinh and Alani 2018, Donis, Gonzalez et al. 2021, Zhou, Xiao and Chen 2024), and Rif1 (Liu, Mei et al. 2018, Mei, Liu et al. 2018, Sad, Mohamed et al. 2021). Similarly, genes down regulated in constant *ph*-KD tumors include known components required for DNA repair and replication fork protection in the presence of replication damage, like the PCNA variant PCNA2 (Feng, Xia et al. 2023) (Table 1). Collectively, misregulation of these genes is expected to lower fork protection, increase DSB formation in response to stalled fork, and impair DSB repair.

**Figure 4:**
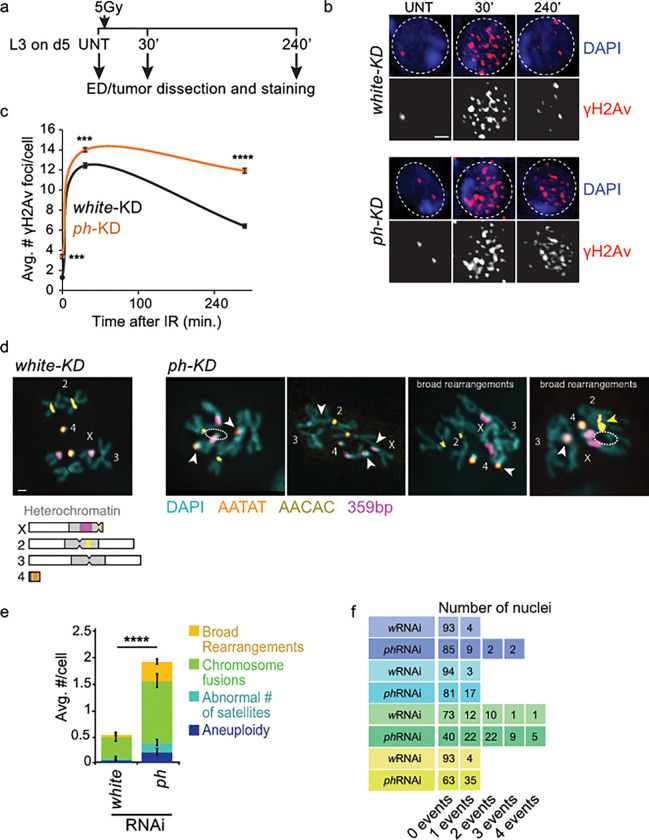
Constant *ph*-KD tumors show delayed recovery after IR induced DSBs. **a)** Schematic representation of the kinetics experiment to assess DSB repair. **b)** Representative images of Drosophila cells from EDs or tumors, stained for γH2Av before (UNT) and at the indicated time points after IR, in *whiteRNAi* (control) and *phRNAi* (constant *ph*-KD) EDs/tumors. Nuclei were stained with DAPI (in blue). **c)** Number of γH2Av foci per cell before (0 min) and after irradiation (30 and 240 min) in *white*-KD (control) and constant *ph*-KD. n≥100 cells representing EDs/tumors from 3 distinct larvae and independent crosses, at the indicated time point. Error bars: SEM. Statistical significance was calculated using a two-sided t-test: ****pval<0.0005. **d)** Examples of karyotypes from *white*-KD (control) and constant *ph*-KD EDs/tumors of female larvae, showing examples of different genomic abnormalities. The scheme of the chromosomes shows the position of the major satellites stained by FISH. Dashed ovals: missing chromosome arms. White arrowheads: fusions. Yellow arrowhead: fusion at the pericentromeric region and abnormal number of satellites. **e)** Quantification of chromosome abnormalities in EDs/tumors in white-KD (control) and constant *ph*-KD. n ≥ 97 karyotypes representing EDs from 3 distinct larvae from independent crosses. Error bars: SEM. Statistical significance was calculated using a two-sided t-test. Scale bars = 1μm.

We directly tested this by investigating DNA break formation through immunofluorescence (IF) analysis of γH2Av foci in tumors dissected from L3 larvae after constant *ph*-KD or in EDs from the temperature-matched *wRNAi* control. Constant *ph*-KD results in a higher number of γH2Av foci in the tissue, indicating a higher level of endogenous DNA damage (Fig. 4a, b). This likely derives from the higher number of replicating cells, which typically experience a higher baseline level of damage than non-replicating cells, along with defective fork protection and repair.

In addition, we investigated the DSB repair response by treating constant *ph*-KD tumors and their controls with 5Gy ionizing radiation (IR), and by quantifying the kinetics of γH2Av focus formation and resolution. Both tumor and ED control tissues showed a significant increase in the number of γH2Av foci 30 min after IR, indicating DSB induction and checkpoint activation. The higher level of repair foci in *ph*-KD tumors relative to the control reflects the higher baseline level of damage. Importantly, constant *ph*-KD tumors display a significantly higher number of γH2Av foci compared to control EDs 4 hours after irradiation, and this difference is much more pronounced than what observed in untreated (UNT) tissues or after 30 min from IR. This indicates that, unlike transient *ph*-KD tumors (Parreno 2024), constant *ph*-KD tumors are defective in DSB repair.

Given the higher amount of DNA damage and defective repair, we hypothesized that constant *ph*-KD tumors might accumulate unrepaired DSBs over time, resulting in chromosome rearrangements and genome instability. We tested this by karyotype analysis of tumors from constant *ph*-KD and EDs from *wRNAi* control in L3 larvae, using pericentromeric fluorescence in situ hybridization (FISH) probes specific for individual chromosomes (Fig. 4d). Remarkably, we observe a 4-fold increase in the frequencies of chromosome rearrangements in constant *ph*-KD tumors relative to the temperature-matched ED controls (Fig. 4d–f). Rearrangements include a large number of chromosome fusions, aneuploidies and abnormal number of satellites (Fig. 4d–f). Moreover, we observe a massive increase in a very rare form of rearrangements characterized by fusions across several chromosomes (“broad rearrangements”), which are so severe that they prevent clear discrimination between the chromosomes (Fig. 4d–f).

In conclusion, tumors induced by PH depletion over 5 days during larval stages are characterized by misregulation of genes required for fork protection and repair, DSB repair defects, and widespread genome instability, which was not observed in EICs derived from transient *ph*-KD.

## Discussion

**Figure 5:**
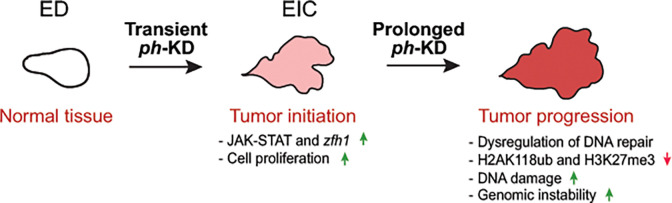
Model for tumor progression in EICs. Upon transient *ph*-KD, EDs switch to a hyper-proliferative cell fate notably due to an irreversible activation of the JAK-STAT pathway and *zfh1* (Parreno 2024). Prolonging *ph*-KD for 4 additional days results in accumulation of more replication damage, misregulation of DNA damage response genes, defective DSB repair leading to persistent DNA damage. This progression reflects a cascade of events where initial hyper-proliferation leads to increased replication stress and subsequent dysregulation of DNA repair mechanisms, ultimately culminating in genome instability.

Although, PRC1 dysregulation is evident in several malignancies, how these tumors acquire altered genome stability remained unclear. Our comparative analysis of epigenetically initiated cancers due to transient *ph*-KD and tumors resulting from constant *ph*-KD offers a rare opportunity to identify progressive changes occurring in a developing tumor. These studies shed light on how epigenetic tumors with a stable genome can quickly transition into a state characterized by massive genomic instability through prolonged PRC1 inactivation (Fig. 5).

We show that, unlike transient *ph*-KD (Parreno 2024), constant *ph*-KD results in loss of H2AK118ub and H3K27me3 at Polycomb target genes, dysregulation of several DNA repair genes, marked defects in DSB repair, and widespread genome instability. Importantly, the transition to a tumor characterized by an unstable genome is reached within 5 days of PH depletion, revealing a rapid acquisition of this typical cancerous phenotype.

However, transient *ph*-KD tumors already display a hyper-proliferating state and some level of misregulation of replication genes. This suggests a progression of the tumor where the hyper-proliferating state is acquired first, resulting in a higher baseline level of damage, followed by dysregulation of fork protection and repair genes (including PRC1 itself), which in turn results in repair defects and chromosome rearrangements. Loss of PRC1 function can contribute to these phenotypes in non-mutually exclusive ways: i) by increasing transcription globally, thus bolstering replication stress form replication-transcription collision (Zeman and Cimprich 2014, Hamperl, Bocek et al. 2017, Gomez-Gonzalez and Aguilera 2019, Chakraborty, Schirmeisen and Lambert 2023); ii) by preventing the establishment of H2AK118ub and H3K27me3 at DSBs, thus interfering with DSB repair (Ismail, Andrin et al. 2010, Campbell, Ismail et al. 2013, Ismail, McDonald et al. 2013, Fitieh, Locke et al. 2021, Fitieh, Locke et al. 2022); and iii) by misregulating the expression of genes required for replication, DNA fork protection and DSB repair, thus increasing the accumulation of unrepaired and mis-repaired breaks. In addition, these defects are amplified in a context of a hyperproliferating tissue, with additional potential for replication damage. Collectively, tumors derived from transient or constant *ph*-KD represent a promising model system to investigate the gradual epigenetic and genomic changes leading to cancer formation.

Together, these observations also highlight the importance of core PRC1 subunits as tumor suppressors and guardians of genome stability. The finding that transient PRC1 depletion leads to epigenetic tumors without inducing genome instability, while prolonged inactivation of this complex results in DNA repair defects and massive rearrangements is also important to inform the strategies for cancer treatment. PRC1 has been considered a potential therapeutic target for cancer (Shukla, Ying et al. 2021, Itoh, Takada et al. 2022, Park, Qin et al. 2023) and our study suggests that PRC1 inactivation will likely increase the sensitivity of tumor cells to DNA damaging agents. On the other hand, such “epi-drugs” may also potentially transform healthy tissues into epigenetically initiated cancers and induce genome instability in response to protracted treatments. Thus, understanding how epigenetic tumors acquire a state characterized by high genome instability is important for establishing improved and safer approaches for cancer therapy.

## Figures and Tables

**Table 1:** List of DNA repair genes dysregulated genes upon constant *ph*-KD. Selected genes from supplementary table 2, their DNA repair function, mammalian homologues and link to cancer. Mismatch Repair (MMR), Base Excision Repair (BER), Nucleotide Excision Repair (NER), Translesion Synthesis (TLS).

Drosophila gene	Mammalian Homologue	Function in DNA repair	Link to cancer

**Up regulated**			

**Mms4**	EME1	Holliday Junction resolvase in complex with Mus81. Replication fork processing and repair.	Overexpressed in several cancers, including colorectal and lung cancer. Associated with poor prognosis and chemo resistance.

**RecQ4**	RECQ4	HR repair	Overexpressed in several cancers, including osteosarcomas, prostate, colorectal, and breast cancers. Associated with poor prognosis and chemo resistance.

**PolH**	POLH	Translesion polymerase	Overexpressed in several cancers, including breast, lung, ovarian and bladder cancers. Associated with poor prognosis and chemoresistance.

**CG43295**	MRNIP	HR repair through phase separation, fork protection	Overexpressed in colorectal cancer, associated with radioresistance and poor prognosis.

**FANCI**	FANCI	Interstrand crosslink repair, stalled fork processing	Overexpressed in several cancers, including lung adenocarcinoma, cervical cancer and liver epatocellular carcinoma, associated with poor cancer prognosis.

**Spel1**	MSH2	Mismatch repair,	Commonly overexpressed in cancer, correlated with poor prognosis in prostate cancer.
**Mlh1**	MLH1	Homeologous recombination	
**Msh6**	MSH6		

**Rif1**	RIF1	Telomere maintenance, DSB repair (prevents resection, promoting NHEJ)	Commonly overexpressed in cancer, promotes drug resistance, correlated with poor prognosis.

**CG10336**	TIPIN	Fork protection complex,	Overexpression induces cancer, promotes drug resistance, associated with poor cancer prognosis.
**timeout/tim2**	TIMELESS	Replication stress response, Homologous recombination repair, Telomere maintenance	

**Claspin**	CLASPIN	Checkpoint activation in response to replication stress	Overexpression induces cancer, promotes radioresistance, associated with poor prognosis.

**Down regulated**			

**PCNA2**	PCNA	Sliding clamp for DNA repair in Drosophila. HR, MMR, NER, BER, TLS,	Downregulated in many cancers, particularly in sarcomas and testicular cancer.

## Data Availability

Data from this study will be available from the corresponding authors upon reasonable request.
